# Reply to Hodges et al. Comment on “Kura et al. Cost-Effectiveness Analysis of Clesrovimab for Respiratory Syncytial Virus in Infants in the United States. *Vaccines* 2026, *14*, 411”

**DOI:** 10.3390/vaccines14090759

**Published:** 2026-08-31

**Authors:** Klodeta Kura, John C. Lang, Dawei Wang, Yoonyoung Choi, Michelle G. Goveia, Anushua Sinha, Yao-Hsuan Chen, Elamin H. Elbasha

**Affiliations:** 1 Health Economic and Decision Sciences, MSD (UK) Limited, London EC2M 6UR, UK; 2 Health Economic and Decision Sciences, Merck Canada Inc., Kirkland, QC H9H 4M7, Canada; 3 Health Economic and Decision Sciences, Merck & Co., Inc., Rahway, NJ 07065, USA; 4 Epidemiology, Merck & Co., Inc., Rahway, NJ 07065, USA; 5 Global Medical and Scientific Affairs, Merck & Co., Inc., Rahway, NJ 07065, USA; 6 Global Clinical Development, Merck & Co., Inc., Rahway, NJ 07065, USA

We thank the authors for their interest in our analysis. We respectfully disagree with several of the characterizations made and address each point below: endpoint alignment, duration of protection, exclusion of non-comparable data sources, and methodological limitations of prior analyses. Our analysis was conducted using rigorous, transparent, and accepted health economic modeling principles, with efficacy inputs derived from global randomized clinical trial (RCT) data.

## 1. Endpoint Alignment Is Necessary When Definitions Differ Across Trials

The authors commented that our base-case analysis relies on a post hoc efficacy endpoint for clesrovimab [1]. The authors argued that the primary efficacy endpoint should have been used as the base case.

Interventions against infectious diseases are frequently observed to have greater efficacy against more severe endpoints [2], a phenomenon which is observed for RSV monoclonal antibodies (mAbs; clesrovimab and nirsevimab) and RSVpreF vaccine [3,4,5,6]. Additionally, there is no gold-standard definition of medically attended lower respiratory infection (MALRI) that is used across pediatric trials aimed at evaluating efficacy of prophylactic mAbs. Thus, ensuring endpoint definition alignment between nirsevimab and clesrovimab was a core principle of our analysis and was a sufficient prerequisite for valid comparisons.

For clesrovimab, the primary efficacy endpoint in the CLEVER clinical trial was *RSV-associated MALRI requiring one or more indicators of lower respiratory infection (LRI) or disease severity* [3]. In contrast, for nirsevimab, the primary endpoint in its trial required *two or more indicators of LRI or disease severity* (i.e., *at least one indicator of LRI and at least one indicator of severity*)—a more severe clinical endpoint [5]. Thus, applying the clesrovimab primary endpoint efficacy estimate in a direct comparison with the nirsevimab primary endpoint would introduce bias in favor of nirsevimab. To enable a valid, like-for-like comparison between clesrovimab and nirsevimab, we adopted clesrovimab efficacy estimates from a post hoc analysis [3] that applied the endpoint of *RSV MALRI requiring two or more indicators of LRI or disease severity* to the CLEVER trial data. This post hoc clesrovimab efficacy endpoint definition was designed to align as closely as possible with the clinical efficacy trials of nirsevimab (which was intended to improve comparability), based on the respiratory signs and symptoms collected in the clesrovimab trial.

Although the authors identify differences between the clesrovimab post hoc efficacy endpoint and the nirsevimab efficacy endpoint (Table 1 of the Comment article [1]), these distinctions are unlikely to be clinically meaningful for the purpose of comparative modeling. Most of the cited differences (grunting, nasal flaring, hypoxic and ventilatory failure) represent overlapping clinical expressions of hypoxemia, the key physiologic construct underlying these indicators, which was assessed and included in the MALRI definitions in both programs.

The same guiding principle, i.e., of ensuring endpoint definition alignment between efficacy estimates, was applied consistently to the comparison of RSVpreF vaccine and clesrovimab.

Economic models aim to estimate value using outcomes that are most relevant to healthcare decision-making, which may not always align with a trial’s prespecified primary endpoint. Consequently, modelers often rely on post hoc analyses, alternative endpoint definitions, extended follow-up data, or indirect treatment comparisons to generate inputs that better address the decision problem. Several published studies have employed such approaches [7,8,9,10].

This model was reviewed and discussed by the Advisory Committee on Immunization Practices (ACIP) Workgroup and subject matter experts, and its outputs were presented at a public ACIP meeting [11].

## 2. Endpoint Selection Reflects Scientifically Appropriate Methodology

The authors stated that modeling guidelines (ISPOR-SMDM, NICE, HAS) require that when multiple plausible inputs exist, sensitivity analyses should test them [1]. They assert that the primary efficacy estimate should have been tested as a scenario and, in the absence of a strong justification, the least favorable option should be the base case.

We agree that modeling guidelines recommend evaluating alternative assumptions through sensitivity analyses. Consistent with these guidelines, we conducted extensive deterministic, probabilistic, and scenario analyses to assess parameter uncertainty, and the conclusions remained robust across these analyses. However, we respectfully disagree with the application of the “least favorable option” principle in this context, as this principle applies only when there is genuine uncertainty about which input is most appropriate. In our case, the primary endpoint and the post hoc endpoint aligned with nirsevimab’s endpoint do not represent alternative estimates of the same quantity—they measure efficacy against endpoint definitions that differ by severity. Thus, the choice of the post hoc endpoint is scientifically appropriate, driven by the need for endpoint alignment, in terms of the clinical outcomes represented. As discussed above, using the primary endpoint efficacy estimate in a comparison with nirsevimab’s efficacy would not represent a conservative assumption; it would represent a methodologically incorrect comparison (i.e., comparing outcomes defined by different clinical criteria).

## 3. Duration Assumptions Are Empirically Grounded in RCT-Demonstrated Protection Periods

The authors stated that (i) the model incorrectly assumed 6 months of protection for clesrovimab and 5 months for nirsevimab, and (ii) the durability claim from day 150 to day 180 relies on only one event per arm.

As described in our publication, the 6-month duration assumption for clesrovimab is directly supported by the CLEVER trial, which followed participants through day 180 and demonstrated sustained protection at that time point [3]. For nirsevimab, the 5-month duration assumption was based on the MELODY trial follow-up period. Additionally, our analysis utilizes sensitivity and scenario analyses where the comparisons were appropriate [12].

1. Probabilistic sensitivity analysis (PSA): 78.5% of simulations were cost-saving; 88% had an ICER below $100,000/QALY (Section 5.4.2 of our original article [12]).
2. Scenario S1.1: Alternative waning function. Clesrovimab remained cost-saving.
3. Scenario S1.2: Equal 5-month duration of protection for both clesrovimab and nirsevimab, with clesrovimab efficacy of 88.0% through day 150 (the aligned post hoc estimate for that time window, within the endpoint-alignment framework). Under this scenario, clesrovimab remained cost-saving relative to nirsevimab (Table 5 of our original article [12]).

We note that the authors cite the HARMONIE trial as evidence for longer nirsevimab durability. We address the HARMONIE trial separately, below.

## 4. Valid Comparisons Require a Uniform Evidence Standard Across All Comparators

There are important scientific reasons why these data sources were not used as primary inputs in our publication.

The HARMONIE trial was not included because its design and endpoints differed from CLEVER and MELODY in scientifically significant ways. Importantly, the data in HARMONIE measured only RSV LRI hospitalizations and, unlike the other studies, reflected seasonality in one specific region (i.e., France, Germany, and UK). For these reasons, HARMONIE was excluded from the primary analysis, consistent with the principle of relying on RCT-based efficacy data with comparable endpoints and populations. Including HARMONIE would have introduced heterogeneity in evidence quality and endpoint definitions that would undermine the comparability of our model inputs.

Real-world effectiveness data for nirsevimab, while valuable for understanding population-level impact, reflect a mix of factors beyond intrinsic product efficacy, including healthcare-seeking behavior, case ascertainment, confounding, and population heterogeneity. Our publication was designed to use RCT-derived efficacy for all comparators to ensure a consistent evidence base. Mixing real-world nirsevimab effectiveness with trial-based efficacy estimates for nirsevimab and clesrovimab would introduce structural bias, because real-world and trial-based estimates differ in design, case ascertainment, and confounding, and applying them unevenly distorts comparative conclusions, that our approach deliberately avoids. We acknowledge that HARMONIE may provide contextual evidence on duration of protection; however, incorporating it into our analysis would introduce heterogeneity for the scientific reasons detailed above. Duration uncertainty is instead addressed through Scenario S1.2, which assigned equal 5-month protection to both interventions and under which clesrovimab remained cost-saving.

## 5. Methodological Limitations in Prior Analyses Undermine Their Comparative Conclusions

The authors state that the prior analyses by Yarnoff et al. [13] and Kieffer et al. [14] used transparent methods and tested scenarios, including a trial-based efficacy scenario, in which nirsevimab remained dominant [13,14].

We have reviewed the Kieffer et al. [14] and Yarnoff et al. [13] analyses in detail and identified several methodological concerns that limit the validity of their conclusions regarding clesrovimab. Specifically, this includes the following:

1. Misestimation of duration of protection:
  - In the base case, Kieffer et al. [14] assumed the period of cumulative efficacy of nirsevimab to be 150 days and limited duration of cumulative efficacy for clesrovimab to 100 days, despite RCT evidence from the CLEVER study demonstrating observed efficacy through day 180 post-dose for all exploratory endpoints [3]. To illustrate how the benefits of clesrovimab were unrealistically minimized, we used data on modeled efficacy against RSV hospitalizations over time from Figure S8 of Kieffer et al. (2025) [14] and calculated the average duration of complete protection (area under the efficacy curve) of nirsevimab and clesrovimab as 157.2 and 88.5 days, respectively (see Supplemental Materials, below).
  - Although the authors considered a scenario with a longer duration of protection of 150 days for clesrovimab, the evidence sources and endpoint definitions were not aligned (as described below). The implied average duration of complete protection of clesrovimab in this less pessimistic scenario is also significantly lower than that of nirsevimab (132 vs. 157.2 days) (see Supplemental Materials, below).
  - Therefore, in both cases, the duration inputs assigned to clesrovimab are shorter than the duration reported in the clesrovimab clinical trial, which in turn reduces the modeled number of RSV events averted by clesrovimab and thereby underestimates its clinical and economic benefit.
2. Mischaracterization of the decay of protection:
  - Based on Kieffer et al. [14] (Figure S8), the modeled protection against RSV-related hospitalization at day 100 post-immunization is 95.3% for nirsevimab and 25.7% for clesrovimab. By day 150, these estimates decline to 59.4% for nirsevimab and 0.3% for clesrovimab.
  - Over the first 150 days post-immunization, the assumed cumulative efficacy against RSV-related hospitalization is 94.2% for nirsevimab and 58.4% for clesrovimab (Supplemental Materials, below).
  - The above are inconsistent with reported RCT data. For example, the reported 150-day efficacy against RSV-related lower respiratory tract infection (LRTI) hospitalization is 80.6% for nirsevimab and 90.9% for clesrovimab [3,4,15,16,17].
3. Misapplication of evidence sources:
  - Kieffer et al. [14] compared real-world effectiveness for nirsevimab against randomized trial-based efficacy for clesrovimab.
  - Although Yarnoff et al. [13] considered a scenario using RCT data for both interventions, the endpoint definitions were not aligned.
  - As described earlier and in our publication, different evidence sourcing introduces structural bias. The correct approach is to follow the gold standard for evidence and use randomized trial-derived efficacy for both clesrovimab and nirsevimab with the most comparable endpoint definitions available.
4. Misalignment of endpoint definitions:
  - Kieffer et al. [14] conducted intervention comparisons using dissimilar endpoints. In their Introduction Section Kieffer et al. state that “clesrovimab comparative efficacy against nirsevimab is unclear due to a lack of data, complicating the evaluation of the practicality and overall value of clesrovimab for widespread use in RSV disease prevention”. However, as previously published and available to Kieffer et al., Zar et al. presented the post hoc analysis of RCT data for clesrovimab as well as a comprehensive analysis of efficacy against a wide range of RSV-associated disease endpoints including mild, moderate and severe disease [3,15].
  - To ensure a balanced comparison that accounts for the relationship between outcome severity and efficacy, endpoint case definitions should be properly aligned across randomized trials. Non-aligned endpoints preclude fair, apple-to-apple comparisons.

Taken together, these methodological choices systematically bias the Kieffer et al. and Yarnoff et al. [13,14] analyses against clesrovimab. Their conclusions regarding the comparative value of nirsevimab should be interpreted in light of these limitations.

## 6. Corroborative Evidence Does Not Require Direct Head-to-Head Validation to Be Scientifically Informative

The authors state that the University of Michigan-CDC model (UM-CDC) cited by Kura et al. is not a direct validation because clesrovimab and nirsevimab were modeled separately with different assumptions [11,18]. We agree that the UM-CDC model is not a direct head-to-head validation, and we did not present it as such. However, when the same UM-CDC model framework was applied to both interventions, each compared against palivizumab, clesrovimab averted more RSV-related events across all categories (outpatient visits, ED visits, hospitalizations, and deaths) and achieved a lower ICER ($104,543/QALY vs. $153,517/QALY), despite a higher per-dose cost ($457 vs. $445) [11,18]. This external corroboration, combined with our own extensive sensitivity and scenario analysis, provides convergent evidence that clesrovimab’s economic advantage over nirsevimab is robust across multiple modeling frameworks.

## 7. Concluding Statement

As demonstrated above, our analysis is grounded in consistent methodological principles such as endpoint definition alignment, RCT-derived efficacy for all comparators, and duration assumptions supported by observed clinical trial data. The use of the post hoc efficacy endpoint for the clesrovimab vs. nirsevimab comparison is scientifically appropriate to enable a valid, endpoint-aligned comparison between two interventions whose clinical trials used different case definitions. This approach is supported by the published CLEVER trial data (Zar et al.) [3].

We have conducted extensive sensitivity and scenario analyses, including equal-duration scenarios, alternative waning functions, and probabilistic sensitivity analyses. There will be limitations inherent in cross-trial comparisons; in these evaluations, clesrovimab remained cost-saving relative to nirsevimab across all scenarios evaluated under our endpoint-alignment framework. All model inputs, assumptions, and scenario analyses are fully documented in our publication and Supplementary Materials. We welcome further scientific dialog and are committed to transparency in our modeling assumptions.

## 8. Supplemental Materials

Here we include supplemental material regarding our review of Kieffer et al.

Modeled efficacy against RSV outcomes over time was based on a sigmoid function and included in Figure S8 of Kieffer et al. [14]. Using embedded data in Figure S8 on efficacy against RSV-related hospitalizations and the following sigmoid function over time *t*, we obtained the values of the parameters as in Table 1 below,

(1) $$VE\left(t\right)=\frac{a0}{1+e^{a1+a2\times t}},$$

where *VE*(*t*) denotes efficacy at a point in time *t*.

**Figure 1:**
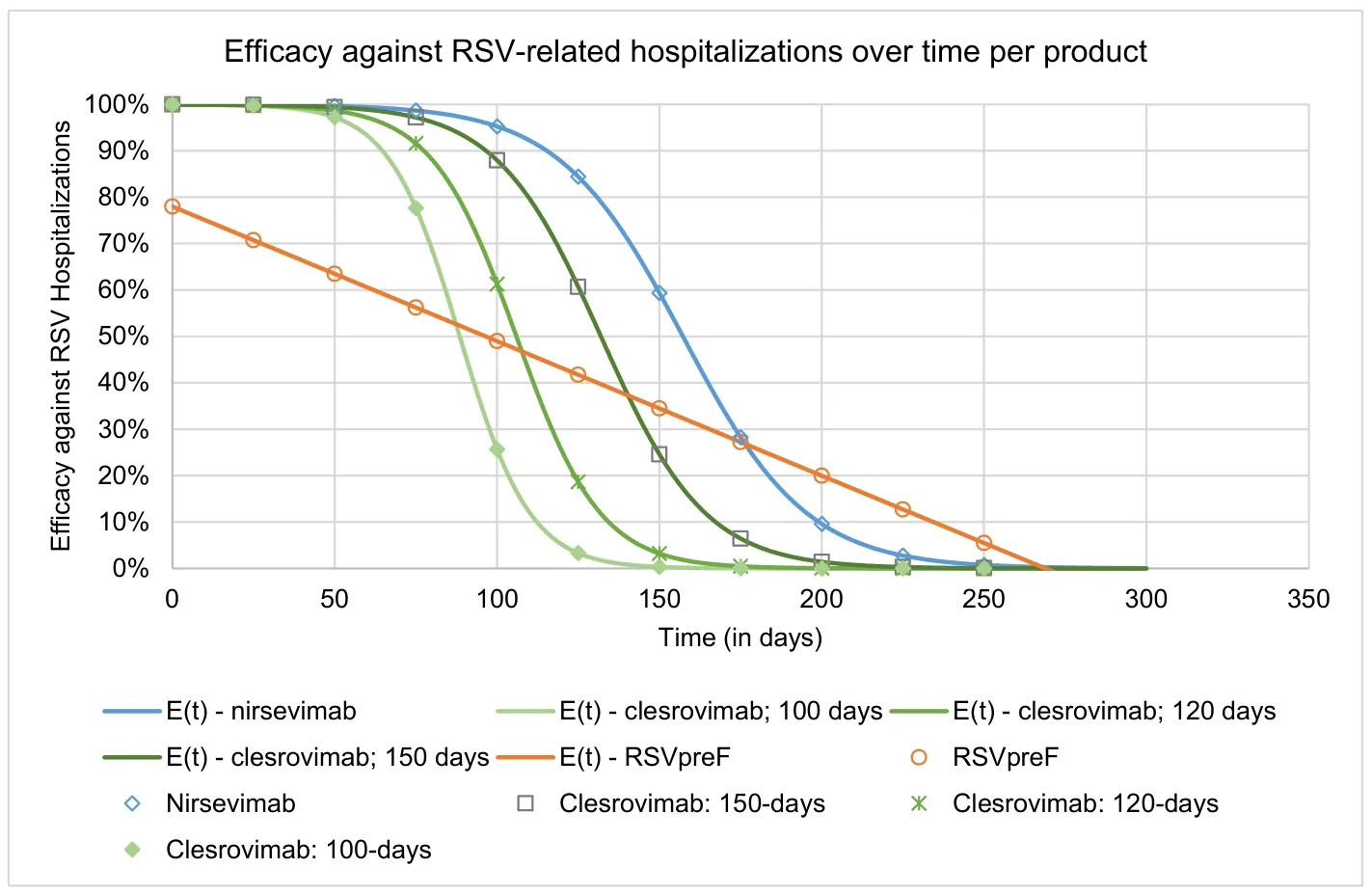
Efficacy against RSV-related hospitalizations over time per product (solid lines are from Figure S8 Kieffer et al.; markers are data-generated using Equation (1)).

To verify that the values in Table 1 were the ones used to construct Figure S8 (Kieffer et al.), we plotted some values using Equation (1) in Figure S8 (reproduced here, Figure 1).

### 8.1. Duration of Complete Protection

The duration of complete protection over a period of *T* days was obtained by integrating *VE*(*t*) in Equation (1):

(2) $$\int_{0}^{T}VE\left(t\right)=\int_{0}^{T}\frac{a0}{1+e^{a1+a2*t}}dt=\frac{a0}{a2}\ln\left[\frac{{\mathrm{e}}^{a2T}\left(1+{\mathrm{e}}^{a1}\right)}{1+{\mathrm{e}}^{a1+a2T}}\right]$$

### 8.2. Cumulative Efficacy

**Figure 2:**
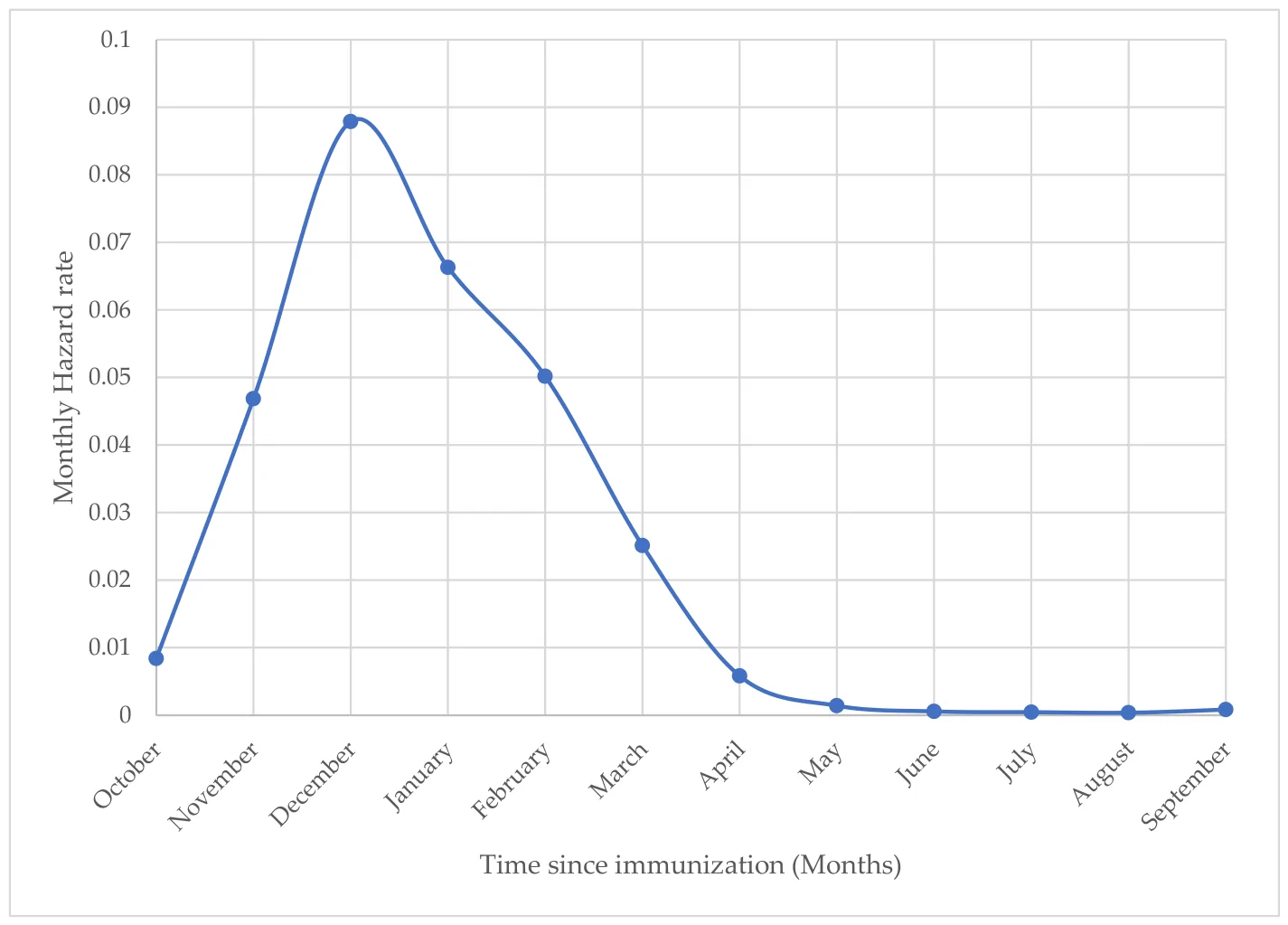
Monthly hazard rate of RSV-related hospitalizations over time since immunization [19,20].

To illustrate the implied cumulative efficacy derived from Equation (1) and compute it with data from clinical trials we first calculate the monthly hazard rate of RSV-related hospitalization *hz* among healthy unimmunized infants born in October as they age (Figure 2). Second, we convert this monthly hazard rate into daily rate and use it with Equation (1) to compute cumulative efficacy over *T* days as

(3) $$1-\frac{1-e^{-\int_{0}^{T}\left[hz\left(\frac{t}{30}\right)/30\right]\left[1-VE\left(t\right)\right]dt}}{1-e^{-\int_{0}^{T}\left[hz\left(\frac{t}{30}\right)/30\right]dt}}.$$

## Figures and Tables

**Table 1:** Parameters and outcomes for modeled efficacy.

*Parameters*	Nirsevimab	Clesrovimab: 100-Days	Clesrovimab: 150-Days
***a0***	1.0	0.999975	1.0
***a1***	–8.24054	–8.17213	–8.20495
***a2***	0.0524101	0.092353	0.0621722
**Outcomes**			
**Duration of complete protection (days)**	157.2	88.5	132.0
***Cumulative efficacy over*** **(%)**			
**100 days**	98.8	80.9	97.3
**150 days**	94.2	58.4	86.7
**180 days**	91.2	55.0	82.4

## Data Availability

The contributions presented in this study are included in the article. Further inquiries can be directed to the corresponding author.
